# Motion Analysis of Ear Surgery Training on an Ovine Model

**DOI:** 10.7759/cureus.109448

**Published:** 2026-05-22

**Authors:** Mara Tanase, Mihai I Tănase, Marcel Cosgarea, Alma A Maniu, Doinel G Rădeanu, Mirela C Stamate, Cristina Blebea, Constantin Stan, Maximilian Dindelegan, Violeta Necula

**Affiliations:** 1 Department of Otolaryngology, Iuliu Hațieganu University of Medicine and Pharmacy, Cluj-Napoca, ROU; 2 Department of ENT, Universitatea "1 Decembrie 1918” din Alba Iulia, Alba Iulia, ROU; 3 Department of ENT, Iuliu Hațieganu University of Medicine and Pharmacy, Cluj-Napoca, ROU; 4 Medical-Pharmaceutical Research Centre, Universitatea "Dunărea de Jos” din Galați, Galati, ROU; 5 Department of Surgery – Practical Abilities, Iuliu Hațieganu University of Medicine and Pharmacy, Cluj-Napoca, ROU

**Keywords:** accelerometer, experimental animal model, myringotomy, otolaryngology training, otosurgery, stapedectomy

## Abstract

Introduction: Motion analysis is crucial for surgical training, especially in intricate procedures like ear surgery. Hand movements during myringotomy and stapedectomy training on a sheep’s head model were evaluated in this study using accelerometer-based sensors. The aim is to identify specific motion characteristics-including tremor and speed consistency-that distinguish surgical expertise levels and quantify the efficiency gap between trainees and experts.

Materials and methods: Participants (n=24) were divided into three groups: residents (n=10), specialists (n=10), and senior surgeons (n=4). Accelerometer sensors captured data on time, path length, acceleration, tremor, and speed consistency. Data analysis utilized the Kruskal-Wallis test followed by Mann-Whitney U post-hoc comparisons with Bonferroni correction to identify specific inter-group differences.

Results: Significant differences were found across all parameters (p < 0.001). Senior surgeons demonstrated the highest efficiency, performing stapedectomy 70.6% faster than residents and 42.9% faster than specialists. Notable gaps in stability were observed; residents exhibited a 14% lower speed consistency and 66.7% higher tremor levels compared to seniors. Specialists occupied an intermediate phase but still maintained a 42.8% higher tremor rate than senior surgeons, indicating a prolonged refinement period for fine motor control.

Conclusions: The data reveal a clear, quantifiable learning curve. Residents require significantly more time and exhibit less controlled movements, while specialists show technical refinement but have not yet reached the high-level economy of motion seen in seniors. These objective metrics, particularly efficiency gaps and stability consistency, can help tailor training programs to focus on precision and economy of motion.

## Introduction

Surgical training is an ever-evolving field, continuously integrating new technologies to enhance the learning process and elevate the acquisition of surgical skills. In this pursuit of improved training methods, motion analysis has emerged as a pivotal tool, offering objective insights into movement patterns to evaluate surgical performance [1]. By yielding quantifiable insights into surgical technique and efficiency, motion tracking enables instructors to pinpoint technical deficiencies and refine training protocols to meet individual learner needs [2]. 

Given that otologic surgery demands exceptional manual dexterity and spatial perception, motion analysis serves as an extremely useful tool for objective skill acquisition in this field [3]. Recognizing the critical role of motion analysis in surgical education, this study delves into the specific challenges posed by ear surgery, focusing on two fundamental procedures: myringotomy and stapedectomy. Specifically, this study evaluates five critical indicators of surgical dexterity: procedure duration, path length, mean acceleration, physiological tremor, and speed consistency [4]. By quantifying these parameters, we can transition from subjective observation to a data-driven definition of surgical proficiency.

Myringotomy, a procedure involving an incision in the tympanic membrane, demands precision and control to avoid complications. Stapedectomy, a delicate operation to improve hearing, necessitates exceptional fine motor skills and a steady hand [5]. By employing a sheep's head model as a surgical training platform, this study aims to capture and analyze the nuanced hand motions involved in these procedures. Beyond identifying broad differences, this research aims to calculate the specific performance gaps that exist between experience levels, providing a numerical benchmark for what constitutes 'expert' efficiency in otologic microsurgery [6]. 

The primary objective of this research is to identify distinctive motion characteristics that differentiate surgeons of varying experience levels, from residents to specialists, and ultimately to senior surgeons. By objectively quantifying these motion features, we strive to enhance the objectivity and effectiveness of training feedback mechanisms. The insights from this research will support the creation of more customized training programs, ultimately leading to improved surgical performance and patient outcomes [7].

## Materials and methods

In our quest to create a robust and informative study, we aimed to simulate realistic surgical conditions while adhering to ethical practices. The study was approved by the Institutional Review Board of the Iuliu Hațieganu University of Medicine and Pharmacy on March 24, 2025 (protocol no: AVZ74/24MAR2025). Following this approval, the study was conducted over a two-day period, specifically on March 27 and March 28, 2025. We employed 24 fresh-frozen adult sheep heads (Native Romanian Turcana breed), obtained from a local slaughterhouse with veterinary clearance, as a substitute for human subjects. 

Participants

In this study focusing on enhancing surgical training through motion analysis, the selection of participants was crucial to representing a range of experience levels. Participants aged 25-54 years were recruited from different hospitals across Romania to ensure diverse perspectives in expertise. Inclusion criteria were defined by the level of ear surgery experience and affiliation with the training center. To ensure the integrity of the motion data, participants were required to have no known medical conditions that promote tremor and could not be undergoing pharmacological treatment that could favor tremor. Accordingly, the study was designed to categorize participants into three separate expertise levels: first-year otorhinolaryngology residents with minimal to no experience in ear surgical procedures, junior otorhinolaryngology specialists with three to five years of experience in ear surgery, and senior otorhinolaryngologists with 20 years or more of experience in ear surgery.

Procedures and equipment

Before the surgical tasks started, a preparatory phase was undertaken. This involved gradually defrosting the sheep heads at room temperature for approximately 14 hours. Subsequently, the outer ear canals of the sheep heads were flushed with isotonic saline as a standard preparatory step. Following this, each participant was assigned to perform specific otologic procedures on an individual ovine specimen. 

Prior to the hands-on phase, all participants received theoretical instruction regarding the procedures they were scheduled to perform. During the actual assessment, they repeated the same maneuvers using standardized equipment and tools. The surgical procedures performed included unilateral myringotomy, a procedure involving a small incision in the tympanic membrane, and unilateral stapedectomy, a microsurgical procedure performed to improve hearing [8].

In this experiment, unilateral stapedectomy was performed by disarticulating the incus and stapes and then removing the stapes bone without the implantation of a prosthesis. The equipment used in the study included a surgical microscope for magnification and illumination, micro-instruments specialized for microsurgery, and the sheep's head model itself. These procedures and equipment were carefully chosen to create a realistic and controlled training environment for the participants, allowing for hands-on experience without the ethical concerns associated with human subjects. The standardized equipment ensured that all participants had access to the same tools, minimizing variability and allowing for focused assessment of their hand motion skills [9].

Motion sensor

Hand movements were recorded and analyzed using a single, compact Bluetooth accelerometer. The device dimensions were 4 cm × 4 cm × 1.5 cm, with a total weight of under 20 grams. The sensor was positioned on the dorsal aspect of the dominant hand of each participant, specifically at the midpoint of the third metacarpal. To secure the sensor in place, it was covered with latex gloves, with the sensor's x-axis oriented toward the proximal aspect of the participant's hand [10].

Integrated with high-precision triaxial accelerometers and gyroscopes, these sensors recorded Cartesian coordinates (x, y, z) at a sampling rate of up to 256 Hz. In this study, physiological tremor was defined as the high-frequency, low-amplitude involuntary oscillations of the hand recorded during the surgical tasks. These micro-movements were captured by the triaxial accelerometer, which allowed for the quantification of stability and motor control by isolating these specific frequency components from the broader procedural movements. Prior to data collection, we calibrated the devices according to the manufacturer's protocol to establish a precise neutral baseline and ensure alignment with the defined movement axes [11].

Data analysis

By processing data captured at a 256 Hz sampling rate, we can perform an objective assessment of surgical performance. This approach enables the quantification of key efficiency metrics, including total procedure time, path length, and acceleration. 

Procedural duration, recorded in seconds (s), represents the total time taken to execute a surgical maneuver and serves as a correlate for surgical experience. Path length quantifies the cumulative distance traveled by the hand in meters or centimeters (m, cm). Finally, acceleration (m/s²) measures the rate of change in hand velocity throughout the task.

Statistical analysis

To analyze the data collected from the accelerometer sensors, we utilized the IBM SPSS Statistics for Windows, version 26.0 (IBM Corp., Armonk, New York, United States). The accelerometer data were collected and transmitted to computer software via Bluetooth 2.0 and imported into CSV (Comma-Separated Values) format. 

To calculate the parameters for analysis, we developed algorithms in Python 3.12.2 (Python Software Foundation, Wilmington, Delaware, United States). For each group of surgeons-senior, specialist, and resident-we computed the mean values and standard deviations for the parameters. To assess the normality of the distribution, we employed the Shapiro-Wilk Test. 

In addition to the Kruskal-Wallis test, post-hoc pairwise comparisons were conducted using the Mann-Whitney U test with a Bonferroni correction to identify specific differences between experience levels. Furthermore, an efficiency gap analysis was performed to calculate the percentage difference in performance metrics between residents, specialists, and senior surgeons, providing a clearer quantitative measure of the learning curve.

Finally, to compare the three surgeon cohorts, we applied a Kruskal-Wallis test to analyze variations in task duration, hand acceleration, and cumulative path length. This non-parametric test was chosen due to the potential non-normal distribution of the data, as we are dealing with a limited sample size and potential outliers. 

## Results

A total of 24 participants were included in the final analysis, consisting of 10 residents (41.7%), 10 specialists (41.7%), and four senior surgeons (16.6%). The participants ranged in age from 25 to 54 years and were recruited from multiple hospitals across Romania.

Myringotomy

In examining the myringotomy results, clear trends emerged among the experience groups. Senior surgeons showcased the most efficient performance, completing the procedure with the least amount of time, the shortest path length, and the lowest acceleration values. This aligns with the expectation that experience cultivates greater efficiency and control in surgical hand movements. 

Conversely, the resident group displayed the highest values across all three parameters, signifying a need for more time, longer hand-traveled distances, and higher acceleration during the myringotomy. This reflects the learning curve in surgical training, where residents are still developing the fine motor skills and precision needed for such procedures. 

Specialists, possessing an intermediate experience level, fell between the residents and senior surgeons in hand motion metrics. This suggests ongoing refinement of their technique toward the efficiency seen in senior surgeons. 

Statistical analysis validated these trends. The Kruskal-Wallis test revealed significant differences between the three groups for all parameters (p<0.001), underscoring the influence of experience level on hand motion efficiency during myringotomy, which is shown in Table 1.

**Table 1:** Myringotomy performance metrics across experience groups Data represented as mean ± standard deviation (SD). The p-values and the H statistic were calculated using the Kruskal-Wallis test. Statistical significance was defined as p < 0.05.

Experience Group	Time (seconds)	Path Length (cm)	Acceleration (m/s^2^)	Tremor (mm)	Speed Consistency (%)	p-value	Test Statistic (H)
Residents (n=10, 41.7%)	42 ± 11	31 ± 9	2.6 ± 0.9	0.45 ± 0.20	70 ± 11	<0.001	18.42
Specialists (n=10, 41.7%)	34 ± 8	23 ± 6	2.2 ± 0.7	0.35 ± 0.15	78 ± 9	<0.001	18.42
Senior Surgeons (n=4, 16.6%)	26 ± 7	18 ± 4	1.7 ± 0.5	0.20 ± 0.10	85 ± 6	<0.001	18.42

Stapedectomy

In the stapedectomy procedure, which demands even greater precision and fine motor skills, group differences were more pronounced. Senior surgeons again exhibited superior performance, completing the procedure with significantly less time, shorter path lengths, and lower acceleration values compared to the other groups. 

Notably, tremor and speed consistency analysis revealed distinct patterns. Residents displayed the highest tremor and least speed consistency, especially during critical steps like stapes removal. This reflects the challenges faced by novice surgeons in maintaining steadiness and control during microsurgery. 

Specialists showed intermediate tremor and speed consistency, indicating continued refinement of their fine motor skills toward the stability seen in senior surgeons. 

**Figure 1:**
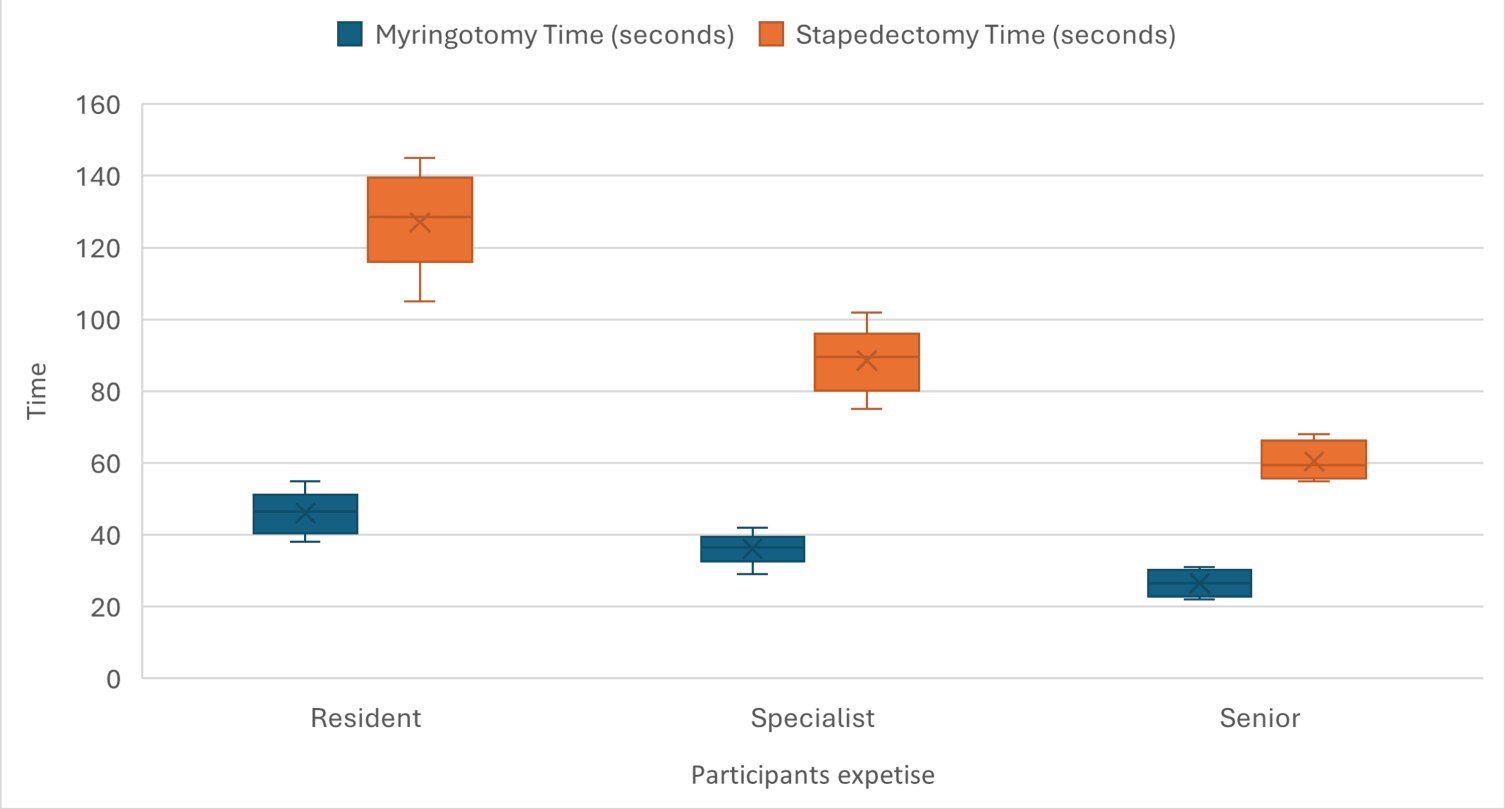
Comparison of myringotomy and stapedectomy procedure times by experience group The box-and-whisker plots represent the distribution of procedural duration in seconds. The central line indicates the median, the 'x' indicates the mean, and the whiskers represent the range of the data.

Statistical analysis confirmed these observations. The Kruskal-Wallis test showed significant differences between the three groups for all parameters (p<0.001), emphasizing the impact of experience on hand motion efficiency during stapedectomy, which is shown in Table 2. A visual comparison of the procedural times for both myringotomy and stapedectomy across the different experience levels is further illustrated in Figure 1.

**Table 2:** Stapedectomy performance metrics across experience groups Data are represented as Mean ± Standard Deviation (SD). The p-values and the H statistic were calculated using the Kruskal-Wallis test, and a value of p < 0.05 was considered statistically significant.

Experience Group	Time (seconds)	Path Length (cm)	Acceleration (m/s^2^)	Tremor (mm)	Speed Consistency (%)	p-value	Test Statistic (H)
Residents (n=10, 41.7%)	115 ± 22	48 ± 12	3.2 ± 1.1	0.75 ± 0.25	68 ± 12	<0.001	19.15
Specialists (n=10, 41.7%)	85 ± 18	37 ± 9	2.7 ± 0.9	0.55 ± 0.22	72 ± 9	<0.001	19.15
Senior Surgeons (n=4, 16.6%)	55 ± 12	22 ± 6	1.9 ± 0.6	0.25 ± 0.15	82 ± 7	<0.001	19.15

When comparing the groups, senior surgeons demonstrated a significantly higher economy of motion in the less complex myringotomy procedure. They completed the task 47.1% faster than residents. To anchor this comparison in absolute values, senior surgeons achieved a mean time of 26 ± 7 seconds, whereas residents required 42 ± 11 seconds to complete the same maneuver. As detailed in Table 3, this 16-second difference highlights the significant efficiency gap and post-hoc significance (p < 0.01) established even in less intricate tasks.

**Table 3:** Pairwise comparison and percentage efficiency gap Data are represented as percentage (%) differences and p-values. Post-hoc significance and the U statistic was determined using the Mann-Whitney U test with Bonferroni correction. A value of p < 0.05 was considered statistically significant.

Metric Comparison	Resident vs. Senior (% Difference)	Specialist vs. Senior (% Difference)	Post-hoc Significance (p-value)	Test Statistic (U)
Myringotomy Time	47.1% Faster	26.7% Faster	p < 0.01	2.0
Stapedectomy Time	70.6% Faster	42.9% Faster	p < 0.001	0.0
Path Length (Total)	48.3% Shorter	23.6% Shorter	p < 0.05	4.5
Tremor Reduction	66.7% Lower	42.8% Lower	p < 0.01	1.5

While specialists are significantly more efficient than residents, they still face a 42.9% efficiency gap in stapedectomy time when compared to senior surgeons. This suggests that while basic competency is reached quickly, the 'refinement toward efficiency' is a prolonged process, as seen in Table 4.

**Table 4:** Stability and consistency analysis

Experience Group	Tremor (mm), mean ± SD	Speed Consistency (%), mean ± SD	Consistency Gap vs. Seniors (% difference)
Residents (n=10)	0.75 ± 0.25	68 ± 12	14%
Specialists (n=10)	0.55 ± 0.22	72 ± 9	10%
Senior Surgeons (n=4)	0.25 ± 0.15	82 ± 7	Reference

## Discussion

This study delves into the interplay between surgical expertise and hand motion efficiency in the domain of ear surgery. By employing motion analysis technology, we sought to uncover the subtle nuances in hand movements, such as tremor and speed consistency, that distinguish surgeons of varying experience levels, from novice residents to seasoned specialists and senior surgeons. Our findings provide quantifiable proof of a distinct learning curve where experience translates directly into greater efficiency, precision, and control [12].

While few studies utilize the ovine model for motion analysis in otosurgery, our results align with broader findings in surgical education. For instance, studies in laparoscopic and orthopedic surgery have similarly demonstrated that motion tracking can distinguish between expertise levels based on path length and economy of movement. Senior surgeons in our study demonstrated superior performance, completing procedures with remarkable speed and dexterity. Their movements were characterized by an economy of motion that minimized unnecessary travel, mirroring findings by Uemura et al. regarding expert vs. novice differentiation in surgical tasks [13].

A critical finding in our study was that senior surgeons exhibited a 66.7% lower tremor rate than residents. While physiological tremor is a common occurrence among surgeons across all age groups, it is often paradoxically expected to increase with age due to natural biological factors. However, in the context of microsurgery, this is frequently offset by the reduction of stress-induced physiological tremor. Novice residents often experience higher autonomic arousal during complex tasks like stapedectomy, which manifests as increased tremor [14].

In contrast, the marked reduction in tremor among senior surgeons reflects more than just physical motor control; it indicates reduced cognitive load and procedural automatization. Because the senior surgeon has reached a level of unconscious competence, their cognitive resources are not exhausted by the mechanics of the procedure, allowing for a steadier hand. This level of stability sets a numerical benchmark for what constitutes expert motor control in otologic microsurgery [15].

Specialists showed a clear progression toward efficiency compared to residents, yet they still face a 42.9% efficiency gap in stapedectomy time and a 10% consistency gap in speed compared to seniors. This suggests that while basic technical competency is acquired during the specialist phase, the mastery of high-level rhythmic consistency and total tremor suppression is a much more prolonged process. The gap exists because specialists, despite their three to five years of experience, may still be refining the technical "flow" of the procedure, whereas seniors have optimized their economy of motion over decades [16].

The integration of motion analysis technology offers a powerful tool for objective assessment and personalized feedback. Starting from this subject, a compelling area for future research would be the investigation of biofeedback-assisted training. Specifically, future studies could explore whether real-time auditory or visual alerts regarding tremor and speed consistency can accelerate the learning curve for residents and specialists, potentially shortening the decade-long gap required to reach senior-level proficiency [17].

The use of the sheep's head model provided a realistic and ethical platform for this training, allowing for the refinement of these specific motor skills without risk to human subjects. While the study has limitations regarding cohort size and geographic scope, the findings highlight the importance of deliberate practice and targeted training in developing surgical expertise. Although motion analysis provides a high-fidelity proxy for technical skill, it is important to note that these metrics represent manual dexterity within a simulated environment and do not account for the multifaceted intraoperative decision-making required in live surgery [18]. Future research should continue to investigate whether the skills developed on such training models effectively transfer to the operating room environment.

## Conclusions

This study has provided valuable insights into the relationship between surgical experience and hand motion efficiency in ear surgery. The findings highlight the importance of deliberate practice and targeted training in developing surgical expertise. The integration of motion analysis technology and the use of realistic training models can significantly enhance surgical education and contribute to improved patient outcomes. While the study has limitations, it underscores the potential of motion analysis as a tool for objective assessment and personalized feedback in surgical training. Future research should investigate whether the skills developed on training models effectively transfer to the operating room and if these findings hold true across broader populations.
